# Draft Genome Sequence of *Alteromonas macleodii* Strain MIT1002, Isolated from an Enrichment Culture of the Marine Cyanobacterium *Prochlorococcus*

**DOI:** 10.1128/genomeA.00967-15

**Published:** 2015-08-27

**Authors:** Steven J. Biller, Allison Coe, Ana-Belen Martin-Cuadrado, Sallie W. Chisholm

**Affiliations:** a Department of Civil and Environmental Engineering, Massachusetts Institute of Technology, Cambridge, Massachusetts, USA; b Departamento de Producción Vegetal y Microbiología, Universidad Miguel Hernandez, Alicante, Spain; c Department of Biology, Massachusetts Institute of Technology, Cambridge, Massachusetts, USA

## Abstract

*Alteromonas* spp. are heterotrophic gammaproteobacteria commonly found in marine environments. We present here the draft genome sequence of *Alteromonas macleodii* MIT1002, which was isolated from an enrichment culture of the marine cyanobacterium *Prochlorococcus* NATL2A. This genome contains a mixture of features previously seen only within either the “surface” or “deep” *Alteromonas* ecotype.

## GENOME ANNOUNCEMENT

*Alteromonas* spp. are copiotrophic bacteria widely distributed throughout the marine environment, where they can play a notable role in the processing of dissolved organic carbon pools (1, 2). The type strain of this group is *Alteromonas macleodii* ATCC 27126, which was isolated from surface waters in Hawaii (3); since then, *Alteromonas* spp. have been obtained from diverse locations, including deep (>1,000 m) waters in the Mediterranean. Genomic analyses have shown that *A. macleodii* strains cluster into two phylogenetic groups, termed the “surface” and “deep” ecotypes, correlated with where they were found in the water column (4). Recently, the deep group was reclassified as a separate species, *Alteromonas mediterranea* (5).

We isolated *A. macleodii* MIT1002 in 2010 from an enrichment culture of the marine cyanobacterium *Prochlorococcus* NATL2A, originally isolated in 1990 from seawater collected at a 10-m depth in the North Atlantic (38°59′N 49°44′W) (6). The enrichment culture had been maintained for more than two decades via serial transfer in sterile natural seawater-based Pro99 medium (7) at 21°C under ~40 µmol photon m^-2^ s^-1^ illumination. As Pro99 does not contain any added organic carbon to support the growth of *A. macleodii* MIT1002, *Prochlorococcus* must have provided this strain with organic carbon and perhaps other nutrients. *A. macleodii* MIT1002 was isolated by streaking a sample of the *Prochlorococcus* NATL2A culture onto a ProMM plate (Pro99 plus lactate, pyruvate, glycerol, acetate, Va vitamins, and 1.4% Noble agar) (8), and allowing cells to grow at room temperature. Following colony purification, the isolate was maintained in liquid ProMM medium.

Genomic DNA libraries were prepared as previously described (9) and sequenced on an Illumina GAIIx, yielding approximately 480,000 200 × 200-nucleotide (nt) paired-end reads. These were overlapped using SHERA (10), with default settings. Low-quality regions of the reads were removed using qualitytrim (version 3.2; CLC bio) and assembled by the Newbler assembler (version 2.6; 454/Roche), with the following parameters: “-e 200 –rip.” We retained contigs of >500 bp.

The draft genome contains 125 contigs with a total length of 4,667,157 bp and an *N*_50_ of 79,162. The total assembly length and G+C content (44.6%) are consistent with those of other sequenced *A. macleodii* isolates (1). MIT1002 has 4,213 protein-coding sequences and 71 RNAs, as predicted by the RAST annotation server (11). Phylogenetic analysis indicates that this strain is a member of the *A. macleodii* surface ecotype, sharing 96.9% average nucleotide identity (12) with two surface ecotype isolates, *A. macleodii* ATCC 27126 and the English Channel 673 strain (1). MIT1002, however, also contains a number of genetic features in common with the *A. mediterranea* DE (deep ecotype) strain, including a set of hydrogenases and metal resistance genes.

This mixture of traits normally associated with either the surface or the deep-water environment raises the possibility that MIT1002 may contain adaptations allowing it to withstand vertical mixing. This provides an intriguing parallel with the distribution and physiology of the *Prochlorococcus* NATL2A strain with which it was coisolated, as NATL2A is relatively abundant during mixing events and also occupies an intermediate position between high and low-light-adapted ecotypes of *Prochlorococcus* (13–15).

### Nucleotide sequence accession numbers.

This whole-genome shotgun project has been deposited at DDBJ/EMBL/GenBank under the accession no. JXRW00000000. The version described in this paper is version JXRW01000000.
